# Specific prediction of mortality by oxidative stress‐induced damage to RNA vs. DNA in humans

**DOI:** 10.1111/acel.13839

**Published:** 2023-05-15

**Authors:** Anders Jorgensen, Ivan Brandslund, Christina Ellervik, Trine Henriksen, Allan Weimann, Per Kragh Andersen, Henrik E. Poulsen

**Affiliations:** ^1^ Psychiatric Center Copenhagen Mental Health Services Copenhagen Denmark; ^2^ Institute of Clinical Medicine, Faculty of Health and Medical Sciences University of Copenhagen Copenhagen Denmark; ^3^ Department of Clinical Immunology and Biochemistry Lillebaelt Hospital Vejle Denmark; ^4^ Faculty of Health Science, Institute of Regional Health Research University of Southern Denmark Odense Denmark; ^5^ Department of Laboratory Medicine, Boston Children's Hospital Harvard Medical School Boston Massachusetts USA; ^6^ Department of Data Support Region Zealand Sorø Denmark; ^7^ Department of Clinical Pharmacology University Hospital Copenhagen, Bispebjerg and Frederiksberg Copenhagen Denmark; ^8^ Section of Biostatistics University of Copenhagen Copenhagen Denmark; ^9^ Department of Cardiology Copenhagen University Hospital Hillerød Hillerød Denmark; ^10^ Department of Endocrinology Copenhagen University Hospital Bispebjerg‐Frederiksberg Copenhagen Denmark

**Keywords:** aging, mortality, nucleic acids, oxidative stress

## Abstract

Modifications of nucleic acids (DNA and RNA) from oxidative stress is a potential driver of aging per se and of mortality in age‐associated medical disorders such as type 2 diabetes (T2D). In a human cohort, we found a strong prediction of all‐cause mortality by a marker of systemic oxidation of RNA in patients with T2D (*n* = 2672) and in nondiabetic control subjects (*n* = 4079). The finding persisted after the adjustment of established modifiers of oxidative stress (including BMI, smoking, and glycated hemoglobin). In contrast, systemic levels of DNA damage from oxidation, which traditionally has been causally linked to both T2D and aging, failed to predict mortality. Strikingly, these findings were subsequently replicated in an independent general population study (*n* = 3649). The data demonstrate a specific importance of RNA damage from oxidation in T2D and general aging.

## Graphical abstract

We studied markers of DNA and RNA oxidation as predictors of death in background and control populations as well as in patients with type 2 diabetes (total *n* = 10,400 persons). We found that in all populations, the RNA marker strongly predicted mortality, whereas the DNA marker did not.

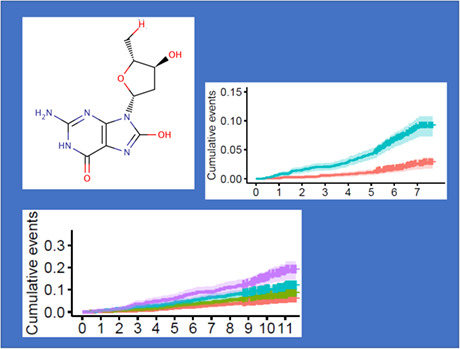

## 1 INTRODUCTION

Damage to nucleic acids from oxidation has been advocated a central mechanism in molecular aging since the first publication of Harman's free radical theory in the 1950s (Campisi et al., 2019; Harman, 1956). On an interspecies scale, there is a clear association between mitochondrial respiration, oxidative modifications to nucleic acids, and life span (Adelman et al., 1988), including in humans (Loft et al., 1994), but longitudinal studies with hard end points are scarce. While the predominant scientific focus has traditionally been on damage to nuclear and mitochondrial DNA (Schumacher et al., 2021), recent studies has uncovered a role for damage to RNA in aging and age‐related disease (Nunomura et al., 2012; Poulsen et al., 2012).

Type 2 diabetes (T2D) is an age‐associated and phenotypically heterogeneous disorder (Nair et al., 2022), in which smoking, elevated glycated hemoglobin levels, elevated blood pressure, and elevated low‐density lipoprotein levels are among the established risk factors for death and cardiovascular events (Rawshani et al., 2018). Oxidative stress has been argued an important contributing mechanism in diabetes (Shah & Brownlee, 2016). We have previously demonstrated in two population studies that the systemic formation of oxidized guanine ribonucleosides, but not oxidized guanine deoxyribonucleosides, was prognostic for death in T2D (Broedbaek et al., 2011; Kjaer et al., 2017). Here, we aimed to (1) test the hypothesis that RNA damage from oxidation is also a predictor of death in nondiabetic controls subjects and the general population, and (2) replicate the finding from T2D patients in an extended cohort.

## 2 METHODS

### 2.1 Cohorts

The Vejle Diabetes Biobank (VDB) study recruited patients with T2D and nondiabetic control individuals aged 25–75 years from 2007 to 2010. Individuals from the Vejle catchment area were classified as patients with diabetes according to criteria using HbA1c values, use of antidiabetic medication, or a registered diagnosis of diabetes in the Danish National Patient Registry, and subsequently refined by individual review. Nondiabetic controls matched by age (in 10‐year intervals) and sex from the same catchment area was invited for participation. Individuals with likely non‐Danish origin (based on the name) was excluded. The total cohort consisted of 2721 T2D patients, 599 type 1 diabetes patients, and 4255 controls. All participants gave various information by questionnaire and interview, and anthropometrics were obtained by physical examination. Blood and urine were analyzed for clinical biochemical analyses by accredited methods at the Vejle Hospital Clinical Biochemistry Laboratory. A biobank of blood, plasma, and urine samples, obtained at the day of examination, was created (Petersen et al., 2016).

The Danish General Suburban Population Study (GESUS) was initiated in January 2010. All individuals >30 years of age and a 25% random sample of individuals aged 20–30 years of the population in the Naestved Municipality, located 70 km south of Copenhagen, Denmark, was invited for participation. Inclusion took place from 2010 to 2013. Information was obtained from questionnaires, physical examination, anthropometrics, bioimpedance, ECG, and clinical biochemical analyses by accredited methods by the Naestved Hospital Clinical Biochemistry Laboratory (Bergholdt et al., 2013). Of the total number of invited individuals (*n* = 49,707), 21,203 individuals (42.7%) gave consent to participate. A random subsample (*n* = 3649) of the participants gave a urine sample that was used for the analysis of the nucleic acid oxidation marker analysis in this study. Note that 208 of the 3649 individuals were classified as patients with T2D. In relation to the confirmation survival analysis of the findings from VDB, we regarded the analysis as a general population control analysis and consequently included all individuals, including the T2D patients.

### 2.2 Analysis of urinary markers of systemic oxidative stress on nucleic acids

Urinary collection took place in a standardized manner with rapid freezing of the sample at −20 degrees Celsius or below in both studies. Analysis of the samples took place at three runs from 2012 to 2016. The urinary nucleic acid oxidation markers 8‐oxo‐7,8‐dihydroguanosine (8‐oxoGuo) and 8‐oxo‐7,8‐dihydro‐2′‐deoxyguanosine (8‐oxodG) are validated markers of systemic oxidative stress on RNA/DNA, respectively (Murphy et al., 2022). The markers do not show diurnal variation (Grew et al., 2014) and are highly stable for 10+ years when stored at −20 degrees Celsius (Henriksen et al., 2021). We recently demonstrated that the storage time did not influence the marker levels in the present cohorts (Jorgensen et al., 2022). The exact subcellular origins of the molecules have not been completely clarified, but the cellular release of 8‐oxodG is thought to stem from enzymatic repair of DNA and/or the nucleotide pool, whereas the release of 8‐oxoGuo is thought to stem from the degradation of RNA. In steady state, the rate‐limiting step in the excretion of the markers is the systemic formation (i.e., the rate of oxidation) rather than the amount of repair/degradation of DNA/RNA; a notion we recently corroborated in an in silico study (Jorgensen et al., 2021).

The determination of the markers was performed with ultra‐performance liquid chromatography–tandem mass spectrometry (UPLC‐MS/MS) using the Acquity UPLC system and Xevo TQ‐S triple quadrupole mass spectrometer purchased from Waters Corp., Milford, USA (Rasmussen et al., 2016). Ultra‐performance liquid chromatography–tandem mass spectrometry is a validated and highly precise method for measuring urinary nucleic acid oxidation products (Barregard et al., 2013). Our method includes internal standards to secure high levels of consistency over time (Henriksen et al., 2021). The spot urine sample analyses of 8‐oxoGuo and 8‐oxodG were corrected for urinary creatinine levels, which were analyzed by the Jaffe method (Henriksen et al., 2009). The urinary excretion values of 8‐oxoGuo and 8‐oxodG are expressed as nmol/mmol creatinine. Data on 8‐oxoGuo and 8‐oxodG excretion vs. mortality for a subset of the VDB T2D patients aged 60+ years (*n* = 1863) and with shorter follow‐up were published previously (Kjaer et al., 2017).

### 2.3 Linkage to Danish register‐based data

All Danish residents are assigned a unique 10‐digit number, the Civil Personal Register (CPR) number. This number makes it possible to link information from a multitude of registers on an individual level. In this study, data obtained from the VDB and GESUS cohorts were used to establish databases held on restricted and logged drives on secure servers at the hospital system of the Capital Region of Copenhagen. Data were transferred to dedicated secure servers at Statistics Denmark, and the CPR number was encrypted to a PNR number for linkage to the Danish National Causes of Death Register (Helweg‐Larsen, 2011), updated to 31 December 2018.

### 2.4 Statistical analysis

The predefined primary outcome in the survival analysis was death from all causes. Kaplan–Meier and cumulative event plots were generated for T2D patients (VDB), controls (VDB), and the background population individuals (GESUS) vs. the RNA and DNA marker, respectively. Cox proportional hazards regression analysis with age as the underlying timescale was performed, in which individuals were followed from age at recruitment to age at end of follow‐up or death. The use of age as the underlying time scale effectively corrects for baseline differences in age between the quartiles since no modeling assumptions are imposed for the relationship between mortality rates and age. In the VDB cohort, we constructed quartiles based on the full dataset to allow for comparisons of the risk of death in T2D patients vs. controls at the same absolute 8‐oxoGuo and 8‐oxodG excretion levels. Because the absolute excretion levels of 8‐oxoGuo and 8‐oxodG differed between the groups, the distribution of individuals at risk within each quartile differs in the two groups. Based on the quartiles, we calculated hazard ratios of all‐cause mortality, using the first quartile of the control population as reference to allow for the simultaneous comparison of mortality risk within and between the T2D/control groups. We also made spline plots of marker levels vs hazard ratios on a continuous scale and performed interaction analyses on the full VDB dataset, using models with and without an interaction term (8‐oxoGuo and 8‐oxodG quartile * diabetes status), subsequently comparing these models using likelihood ratio tests. With respect to the GESUS cohort, due to the fewer deaths and shorter follow‐up, we constructed categories of low vs. high 8‐oxoGuo and 8‐oxodG levels based on the median of the datasets. The Cox proportional hazard regression models were adjusted for age (by using it as baseline time scale) and sex (Model 1), and Model 2 further adjusted for smoking status, body mass index, glycated hemoglobin, calculated glomerular filtration rate, and plasma C‐reactive protein concentration. A range of exploratory analyses stratifying for combinations of age group (over and under 60 years) and sex were performed. Finally, exploratory analyses of the correlation between age and 8‐oxodG/8‐oxoGuo excretion per calculated basal metabolic rate (BMR) (calculated by Mifflin et al's formula (Mifflin et al., 1990)) was performed by linear regression models, using log‐transformed 8‐oxo marker/BMR values adjusted for sex.

All analyses and plots were made in R (Anon, 2021) with the packages “data. Table,” “survival,” “Publish,” “ggplot2,” “survminer,” and “Greg” installed.

### 2.5 Ethics

The cohort studies and the subsequent analysis of the urinary markers were approved by the local ethics committees (Vejle biobank: S‐20080097, amendment protocol 37831; GESUS Biobank: SJ‐113, SJ‐114) and reported to the Danish Data Protection Agency. All participants of VDB and GESUS gave written informed consent before inclusion.

## 3 RESULTS

Sociodemographic and biochemical data for the T2D patients and nondiabetic controls from the VDB cohort are presented in Table S1. Cross‐sectional levels of both nucleic acid oxidation markers were higher in T2D vs controls (8‐oxoGuo (mean (sd)): T2D = 3.0 (1.2), controls = 2.2 (0.8), *p* < 0.001. 8‐oxodG: T2D = 1.8 (0.8), controls = 1.6 (0.7), *p* < 0.001) (Table S1). The 8‐oxodG and 8‐oxoGuo excretion was highly correlated (Pearson's correlation coefficient = 0.59, *p* < 2.2 × 10^−16^).

**FIGURE 1:**
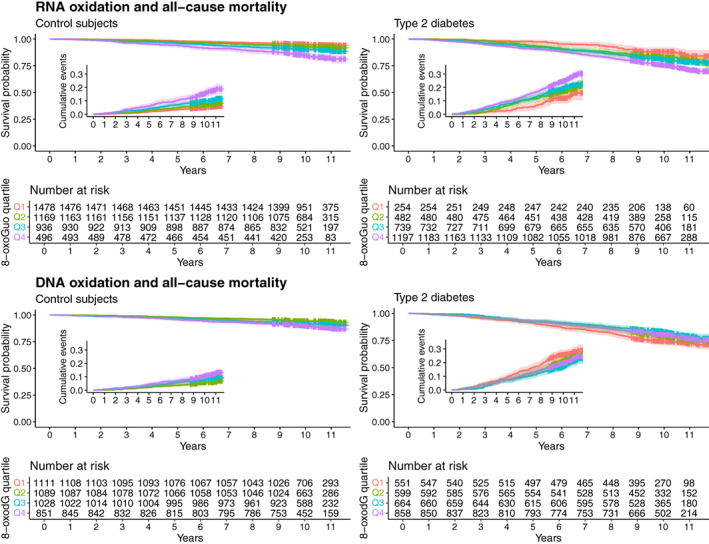
Kaplan–Meier and cumulative event plots (insert) of all‐cause mortality vs. quartile of urinary RNA (8‐oxoGuo, upper panels) and DNA (8‐oxodG, lower panels) oxidation marker excretion in type 2 diabetes (right panels) and healthy controls (left panels). The 8‐oxoGuo and 8‐oxodG excretion quartiles were derived from the full dataset, that is, including both type 2 diabetics and controls.

**FIGURE 2:**
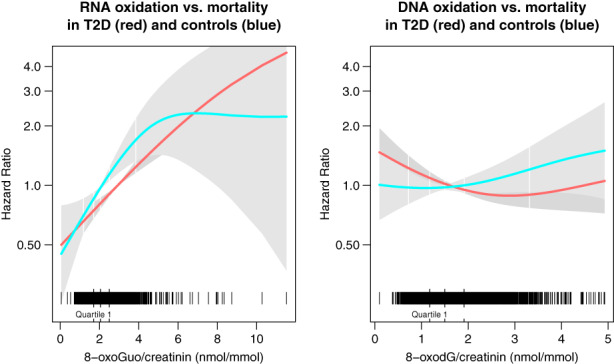
Spline plots for urinary RNA and DNA oxidation marker excretion vs. all‐cause mortality in the VDB cohort. The plots depict continuous levels of the creatinine‐corrected 8‐oxoGuo and 8‐oxodG levels vs. hazard ratio for all‐cause mortality. The black panel indicates the distribution of data points at a given marker level.

Survival and cumulative event curves of all‐cause mortality by quartiles of baseline 8‐oxoGuo and 8‐oxodG marker levels are presented in Figure 1, and the corresponding hazard ratios (HR) for mortality are presented in Table 1. In both T2D and controls, the risk of mortality increased sequentially by each increasing quartile of 8‐oxoGuo excretion, with the highest risk seen in Q4 of the T2D group (HR = 3.38 [2.62;4.36], *p* < 0.0001). HRs for mortality was similar in Q4 of the nondiabetic controls (HR = 2.06 [1.50;2.84], *p* < 0.0001) vs. Q1 in T2D patients (HR = 1.97 [1.31;2.95], *p* < 0.0001). The increases in HR followed the same overall pattern in the T2D and control groups as evidenced by a nonsignificant test for interaction. In contrast, there was no sequential increase in HRs by increasing quartiles of 8‐oxodG, which in fact nominally decreased in Q2 and Q3 in both the control and T2D groups (Table 1). The findings were robust to the full adjustment, and the pattern was confirmed by spline plots of continuous marker levels vs. hazard ratios for all‐cause mortality, showing a mostly linear association between 8‐oxoGuo excretion and mortality vs. a U‐shaped association between 8‐oxodG excretion and mortality, where lower and higher levels were associated with a slightly higher mortality risk (Figure 2). The results did not materially change when stratifying by combinations of age group and sex, but indicated that the association between 8‐oxoGuo and mortality was more pronounced in younger individuals (<60 years) (Table S2).

**TABLE 1:** All‐cause mortality vs. quartile of urinary RNA (8‐oxoGuo) and DNA (8‐oxodG) oxidation marker excretion in type 2 diabetes and healthy controls from the Vejle Diabetes Biobank (VDB) cohort.

	Quartile	*N*	Model 1			Model 2		
			HR	95% CI	*p*	HR	95% CI	*p*
8‐oxoGuo								
Control	Q1	1478	1 (Ref)			1 (Ref)		
	Q2	1169	1.06	[0.78;1.45]	0.696	1.10	[0.80;1.49]	0.564
	Q3	936	1.39	[1.03;1.88]	0.034	1.42	[1.05;1.92]	0.025
	Q4	496	2.06	[1.50;2.84]	<0.001	2.15	[1.56;2.96]	<0.001
Type 2 diabetes	Q1	254	1.97	[1.31;2.95]	0.001	1.64	[1.08;2.50]	0.021
	Q2	482	2.48	[1.82;3.37]	<0.001	1.95	[1.40;2.72]	<0.001
	Q3	739	2.47	[1.87;3.26]	<0.001	2.08	[1.53;2.84]	<0.001
	Q4	1197	3.38	[2.62;4.36]	<0.001	2.93	[2.20;3.90]	<0.001
8‐oxodG								
Control	Q1	1111	1 (Ref)			1 (Ref)		
	Q2	1089	0.65	[0.48;0.90]	0.010	0.70	[0.51;0.97]	0.0298
	Q3	1028	0.94	[0.70;1.26]	0.674	0.97	[0.72;1.30]	0.8413
	Q4	851	1.12	[0.84;1.50]	0.430	1.14	[0.85;1.53]	0.3744
Type 2 diabetes	Q1	551	2.62	[2.02;3.42]	<0.001	1.97	[1.46;2.64]	<0.001
	Q2	599	2.15	[1.64;2.81]	<0.001	1.79	[1.33;2.42]	<0.001
	Q3	664	1.71	[1.30;2.24]	<0.001	1.48	[1.10;2.00]	0.0101
	Q4	858	1.89	[1.47;2.44]	<0.001	1.66	[1.25;2.20]	<0.001

*Note*: The 8‐oxoGuo/8‐oxodG excretion quartiles were derived from the full dataset, that is, including both type 2 diabetics and controls. Results are given as hazard ratios, 95% confidence intervals, and *p*‐values from the Cox proportional hazard regression model, using age as the underlying timescale and the control Q1 category as reference. Model 1 is adjusted for sex; Model 2 is adjusted for sex, smoking status, body mass index, glycated hemoglobin, calculated glomerular filtration rate, and plasma C‐reactive protein concentration.

**FIGURE 3:**
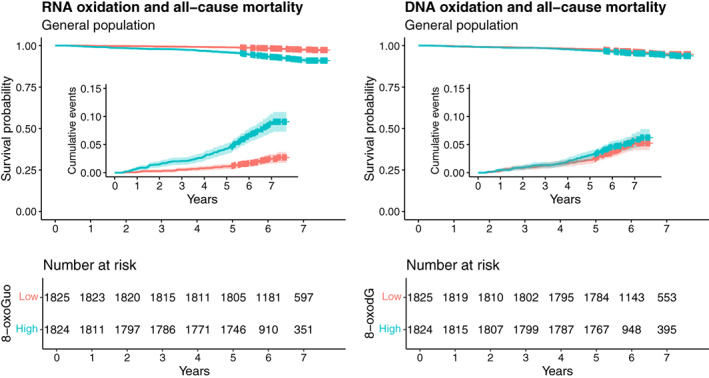
Kaplan–Meier and cumulative event plots of urinary RNA and DNA oxidation marker excretion vs. all‐cause mortality in general population individuals from the GESUS cohort. High vs. low 8‐oxoGuo and 8‐oxodG excretion categories are defined by the medians of the respective datasets.

We subsequently validated the findings in an independent Danish general population cohort (GESUS; *n* = 3649) (Bergholdt et al., 2013). Sociodemographic and biochemical baseline data from this cohort are presented in Table S3. Due to shorter follow‐up and fewer deaths in GESUS, the 8‐oxoGuo and 8‐oxodG levels were categorized as high or low based on the median of the datasets. The same adjustment models as used in the VDB dataset were applied. In GESUS, high vs. low excretion levels of 8‐oxoGuo were strongly predictive of all‐cause mortality, both before and after adjustment (HR_adj_ = 1.71 [1.13;2.59], *p* = 0.011), whereas levels of 8‐oxodG were not (HR_adj_ = 0.95 [0.68;1.34], *p* = 0.783) (Figure 3 and Table 2).

**TABLE 2:** All‐cause mortality vs. quartile of urinary RNA (8‐oxoGuo) and DNA (8‐oxodG) oxidation marker excretion in type 2 diabetes and healthy controls from the Danish General Suburban Population Study (GESUS) cohort.

	Level	Model 1			Model 2		
		HR	95% CI	*p*	HR	95% CI	*p*
8‐oxoGuo	Low	1 (Ref)			1 (Ref)		
	High	1.67	[1.13;2.47]	0.010	1.71	[1.13;2.59]	0.011
8‐oxodG	Low	1 (Ref)			1 (Ref)		
	High	0.98	[0.72;1.34]	0.911	0.95	[0.68;1.34]	0.783

*Note*: The 8‐oxoGuo/8‐oxodG excretion quartiles were defined by the medians of the datasets. Results are given as hazard ratios, 95% confidence intervals, and *p*‐values from the Cox proportional hazard regression models, using age as the underlying time scale. Model 1 is adjusted for sex; Model 2 is adjusted for sex, smoking status, body mass index, glycated hemoglobin, calculated glomerular filtration rate, and plasma C‐reactive protein concentration.

Because the formation of reactive oxygen species predominantly occurs during mitochondrial respiration, oxidative modifications of guanine ribonucleosides and deoxyribonucleosides per consumed oxygen amount can be considered a measure of how well cells control oxidative stress. We thus finally explored 8‐oxoGuo and 8‐oxodG levels per basal metabolic rate (BMR) as a function of age in controls and T2D patients, dichotomized by sex, from the VDB cohort (Figure S4). We found that both 8‐oxoGuo/BMR and 8oxo‐dG/BMR ratio was about twofold higher at age 80 years versus at age 20 years. The 8‐oxoGuo/BMR ratio was increased across the life span in T2D vs. controls in both males and females and followed the same pattern of increase in both groups, as evidenced by a nonsignificant test for interaction. 8‐oxodG/BMR followed the same pattern of increase but was not influenced by diabetes status (Table S5).

## 4 DISCUSSION

We confirmed our hypothesis that the excreted level of the oxidized ribonucleoside, but not the oxidized deoxyribonucleoside, predicts death, not only in T2D as previously demonstrated (Broedbaek et al., 2011; Kjaer et al., 2017), but also in healthy controls and the general population. To our knowledge, this is the first study to demonstrate this phenomenon. The findings were robust to the correction for known modifiers of oxidative stress, including average blood glucose levels as measured by glycated hemoglobin. Furthermore, the marker levels corrected for basal metabolic rate followed the same pattern of increase with increasing age in T2D and controls. Collectively, the findings indicate that the systemic oxidation of RNA is involved in both T2D and general aging by a mechanism independent of glucose dysregulation, and thus may not be merely an epiphenomenon.

The molecular events that dissociate the oxidative modification of the guanine ribonucleoside from the deoxyribonucleoside are only beginning to be revealed. The distribution of guanine oxidation in DNA, RNA, and the nucleotide pool, respectively, is undetermined (Henderson et al., 2010). RNA is more susceptible to oxidative modifications than DNA (Hofer et al., 2005), and—due to the subcellular location of RNA near the mitochondria that produce the H_2_O_2_ capable of oxidizing the guanine moiety through the Fenton reaction—it is potentially more sensitive to mitochondrial dysfunction than DNA. While DNA undergoes extensive and specific enzymatic repair with redundancy in capacity, there is no known repair of RNA, although alternative mechanisms to escape oxidative RNA damage is increasingly being uncovered (Ishii & Sekiguchi, 2019).

What RNA‐specific downstream consequences of oxidative stress could be involved in T2D and general aging? Oxidation of guanine in RNA changes the preferences of base pairing at the ribosome (Thomas et al., 2019) and leads to reduced protein expression levels and malfunctional proteins (Nunomura et al., 2017), which are also features of aging (Martinez‐Miguel et al., 2021). Oxidation of microRNA species can change their regulatory properties, thereby potentially altering function of the target cell. MicroRNA signaling has been implicated in both T2D and aging (Vezza et al., 2021; Wu et al., 2021). For example, reduced expression of microRNA‐21 in pancreatic β cells was recently shown to promote glucose intolerance through downregulation of the GLUT2 transporter (Liu et al., 2022). We thus speculate that higher overall levels of oxidative stress on RNA could affect both the protein fidelity and microRNA regulation pathways, and through these potentially be causally involved in T2D pathogenesis and accelerate aging in both diabetic and nondiabetic individuals.

As mentioned in the Methods section, the exact subcellular origins of the 8‐oxodG and 8‐oxoGuo urinary markers have not been clarified. For 8‐oxodG, base excision repair by, for example, oxoguanine glycosylase 1 is an unlikely source, because the excised product is 8‐oxoguanine rather than 8‐oxodG (Evans et al., 2010). However, both on an organismal and in vitro level, 8‐oxodG has been extensively validated by us and others as a marker of oxidative stress on DNA and the nucleotide pool (Deng et al., 1998; Hu et al., 2015; Lam et al., 2012; Poulsen et al., 2014), as recently discussed in detail elsewhere (Jorgensen et al., 2021). While the same experimental validation for 8‐oxoGuo as a marker of RNA damage has not been performed, there is general consensus that due to its chemical structure, it can only be regarded as a product of systemic RNA oxidation (Weimann et al., 2002). Both markers can be detected in human urine alongside alternative markers of oxidative and nitrative stress (Martinez‐Moral & Kannan, 2022). The organ system(s) that contribute the most to the urinary excretion of the 8‐oxodG and 8‐oxoGuo markers cannot be determined, and the current recommendation is that the markers are best used in diseases or conditions that involve multiple organ systems, such as both T2D and aging per se do (Murphy et al., 2022). We have previously demonstrated that the markers show associations with, for example, iron metabolism (Cejvanovic et al., 2018), statin use and inflammation (Sorensen et al., 2019), as well as hyperthyroidism (Larsen et al., 2021).

In conclusion, we found that the formation of oxidized guanine ribonucleosides predicts death from all causes in both T2D, nondiabetic control subjects and the general population, as measured in two independent Danish cohorts. This extends our previous findings in T2D and thus appears to be a general phenomenon that adds support for the free radical theory of aging. It is, however, at odds with the predominant concept of DNA oxidation as the key event in molecular aging.

## AUTHOR CONTRIBUTIONS

I.B. initiated the Vejle Diabetes Biobank study, C.E. initiated the GESUS study. T.H. and A.W. performed the oxidative stress marker measurements. A.J. and H.E.P. had full access to all data and performed the statistical analyses with input from P.K.A. A.J. and H.E.P. drafted the manuscript. All authors contributed to the concept and design of the study, interpretation of data, and critically revised the manuscript. All authors were fully responsible for the decision to submit for publication. H.E.P. is the guarantor of this work and takes responsibility for the integrity of the data and the accuracy of the data analysis.

## CONFLICT OF INTEREST STATEMENT

None declared.

## Supporting information


Appendix S1.

## Data Availability

Due to restrictions on the access to data stored at Statistics Denmark, raw data from this study cannot be openly shared and only data in groups with >5 observations can be exported from the secure servers. Non‐Danish researchers can get access to individual‐level data by collaboration with Danish researchers through access from computers at approved Danish research institutions.
