# Supplementary material for: Specific prediction of mortality by oxidative stress‐induced damage to RNA vs. DNA in humans
Source: Aging Cell. 2023 May 15;22(6):e13839. doi: 10.1111/acel.13839 (PMC10265158; doi:10.1111/acel.13839)
Supplement: Supplementary file 1 — Appendix S1. [file ACEL-22-e13839-s001.pdf]

Specific Prediction of Mortality by Oxidative Stress-Induced Damage to RNA vs. DNA in Humans with and without Type 2 Diabetes

Supplementary material

Contents:

- Table S1: Baseline data for the VDB cohort
- Table S2: Cox proportional hazard models of RNA and DNA oxidation marker excretion vs. mortality, stratified by age group and sex
- Table S3: Baseline data for the GESUS cohort
- Figure S4: Relationship between urinary RNA and DNA oxidation marker excretion per basal metabolic rate vs. age in the VDB cohort
- Table S5: Regression models for urinary RNA and DNA oxidation marker excretion per basal metabolic rate vs. age in the VDB cohort

Variable	Level	Controls (n=4,079)	Type 2 diabetes (n=2,672)	p-value
**8-oxoGuo (nmol/mmol creatinine)**	Mean (sd)	2.2 (0.8)	3 (1.2)	<0.001
**8-oxodG (nmol/mmol creatinine)**	Mean (sd)	1.6 (0.7)	1.8 (0.8)	<0.001
**Age (years)**	Mean (sd)	59.5 (10.5)	63.3 (8.7)	<0.001
**Sex**	Female (%)	1,825 (44.7)	1,031 (38.6)	<0.001
	Male (%)	2,254 (55.3)	1,641 (61.4)	
**Smoking**	Never (%)	1,601 (39.2)	896 (33.5)	<0.001
	Previous (%)	1,634 (40.1)	1,215 (45.5)	
	Daily (%)	680 (16.7)	457 (17.1)	
	Occasional (%)	153 (3.8)	98 (3.7)	
	Unknown (%)	11 (0.3)	6 (0.2)	
**BMI (kg/m^2)**	Mean (sd)	22.6 (3.9)	26.2 (4.9)	<0.001
**HbA1c (%)**	Mean (sd)	5.5 (0.3)	7 (1.1)	<0.001
**C-reactive protein (mg/L)**	Mean (sd)	2.6 (5.1)	3.7 (6.7)	<0.001
**eGFR (mL/min/1.73 m^2)**	Mean (sd)	78.7 (16.1)	80.4 (18.5)	<0.001

Table S1: Baseline data for control subjects, type 2 diabetics, and the total population of the Vejle Diabetes Biobank cohort. Data are given as means and standard deviations (sd) or absolute numbers and percent and are compared with analysis of variance or chi-square tests, as appropriate.

8-oxoGuo							
		Females < 60 years (N=1290)			*Females > 60 years (N=1565)*		
	**Quartile**	**HR**	**95% CI**	**p**	**HR**	**95% CI**	**p**
**Control**	Q1	1 (Ref)			1 (Ref)		
	Q2	2.33	[0.55;9.81]	0.25055	0.61	[0.25;1.51]	0.2855
	Q3	3	[0.70;12.85]	0.13838	1.21	[0.57;2.60]	0.6159
	Q4	3.66	[0.72;18.78]	0.11921	1.79	[0.84;3.78]	0.1287
**Type 2 diabetes**	Q1	3.21	[0.33;30.91]	0.31223	2.91	[0.88;9.62]	0.0792
	Q2	3.71	[0.61;22.48]	0.15447	2.08	[0.88;4.94]	0.0972
	Q3	7.32	[1.80;29.86]	0.00548	1.5	[0.65;3.48]	0.3439
	Q4	11.44	[3.26;40.12]	< 0.001	2.41	[1.15;5.05]	0.0198
		Males < 60 years (N=1308)			*Males > 60 years (N=2585)*		
**Control**	Q1	1 (Ref)			1 (Ref)		
	Q2	1.32	[0.50;3.50]	0.58013	1.12	[0.78;1.61]	0.54314
	Q3	1.36	[0.37;4.99]	0.64711	1.37	[0.95;1.99]	0.08982
	Q4	3.88	[1.18;12.72]	0.02547	1.92	[1.28;2.88]	0.00172
**Type 2 diabetes**	Q1	2.75	[0.88;8.60]	0.08107	1.34	[0.81;2.22]	0.25325
	Q2	2.43	[0.84;6.99]	0.09978	1.77	[1.19;2.63]	0.00495
	Q3	3.38	[1.33;8.57]	0.01026	1.92	[1.33;2.78]	< 0.001
	Q4	5.62	[2.24;14.11]	< 0.001	2.57	[1.82;3.62]	< 0.001
**8-oxodG**							
		Females < 60 years (N=1290)			*Females > 60 years (N=1565)*		
**Control**	Q1	1 (Ref)			1 (Ref)		
	Q2	1.33	[0.29;5.98]	0.71196	0.76	[0.36;1.60]	0.4746
	Q3	1.35	[0.30;6.09]	0.6969	0.7	[0.35;1.40]	0.3109
	Q4	1.92	[0.42;8.71]	0.39643	1.07	[0.56;2.04]	0.8422
**Type 2 diabetes**	Q1	7.77	[1.70;35.47]	0.00812	2.26	[1.11;4.62]	0.0249
	Q2	9.04	[2.17;37.63]	0.00247	1.64	[0.80;3.37]	0.1769
	Q3	1.28	[0.12;13.55]	0.83532	1.61	[0.80;3.24]	0.1781
	Q4	5.4	[1.21;24.20]	0.02748	1.44	[0.76;2.75]	0.2659
		Males < 60 years (N=1308)			*Males > 60 years (N=2585)*		
**Control**	Q1	1 (Ref)			1 (Ref)		
	Q2	1	[0.30;3.32]	0.99427	0.65	[0.44;0.96]	0.02919
	Q3	1.79	[0.60;5.27]	0.29429	0.98	[0.69;1.40]	0.93136
	Q4	2.7	[0.86;8.49]	0.09002	1.03	[0.71;1.48]	0.88225

**Type 2 diabetes**	Q1	3.76	[1.29;10.93]	0.01518	1.65	[1.16;2.35]	0.00533
	Q2	3.19	[1.03;9.90]	0.04483	1.57	[1.10;2.26]	0.01315
	Q3	2.53	[0.82;7.78]	0.10502	1.37	[0.96;1.96]	0.08432
	Q4	5.12	[1.80;14.55]	0.00218	1.48	[1.05;2.08]	0.02693

Table S2: Cox proportional hazard models of RNA and DNA oxidation marker excretion vs. mortality, stratified by age group and sex. All data are from the fully adjusted models (Model 2, see Statistics section)

Variable	Level	Total
**8-oxoGuo (nmol/mmol creatinine)**	Mean (sd)	2.4 (1.2)
**8-oxodG (nmol/mmol creatinine)**	Mean (sd)	1.9 (1)
**Sex**	F	2,158 (59.1)
	M	1,491 (40.9)
**Age (years)**	Mean (sd)	57.5 (13.1)
**Smoking**	No	2,976 (82.6)
	Yes	625 (17.4)
	Unknown	48
**BMI (kg/m^2)**	Mean (sd)	26.7 (4.6)
**HbA1c (mmol/mol)**	Mean (sd)	37.9 (6)
**C-reactive protein (mg/L)**	Mean (sd)	2.8 (5.8)
**eGFR (mL/min/1.73 m^2)**	Mean (sd)	86.4 (16.1)

Table S3: Baseline data for the 3,649 general population participants with available urine samples from the General Suburban Population Study (GESUS). Data are given as means and standard deviations (sd) or absolute numbers and percent.

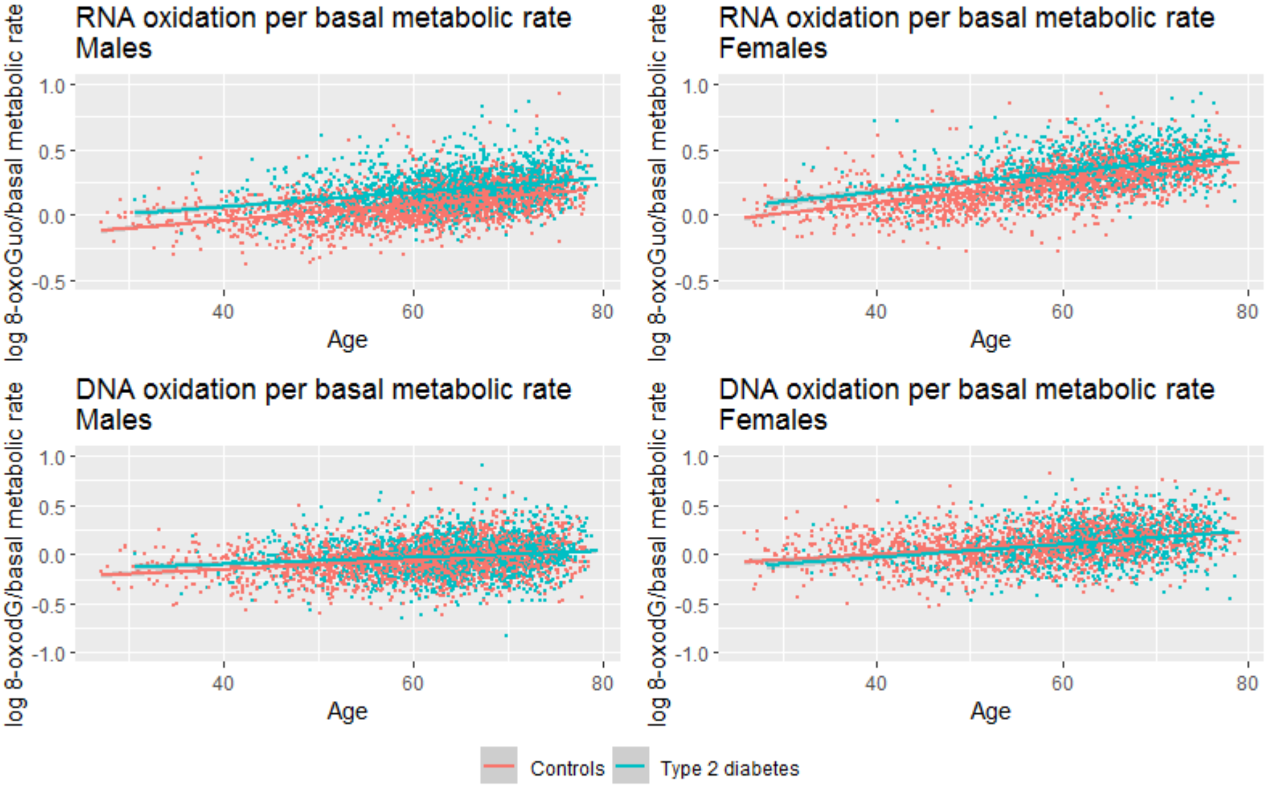

Figure S4: Relationship between urinary RNA and DNA oxidation marker excretion per basal metabolic rate vs. age in the VDB cohort. Data are shown as individual data points of log-transformed marker values per basal metabolic rate (see Methods) vs. age, in males and females, respectively.

8-oxoGuo/BMR	estimate	Std. error	p-value
Age	0.007	0.0001	<0.0001
Diabetes (ref=controls)	0.124	0.022	<0.0001
Sex (ref=females)	-0.167	0.003	<0.0001
Age*diabetes interaction	-0.0006	0.0004	0.071
**8-oxodG/BMR**	**estimate**	**Std. error**	**p-value**
Age	0.005	0.0002	<0.0001
Diabetes (ref=controls)	0.024	0.030	0.434
Sex (ref=females)	-0.160	0.004	<0.0001
Age*diabetes interaction	-0.0002	0.0004	0.645

Table S5: Regression models for log-transformed 8-oxoGuo and log-8-oxodG per basal metabolic rate (BMR) vs age, diabetes status (controls vs. T2D), sex and age by diabetes status interaction in 6,751 individuals from the Vejle Diabetes Biobank cohort. See Methods for the calculation of BMR.
